# Mapping current research and identifying hotspots on mesenchymal stem cells in cardiovascular disease

**DOI:** 10.1186/s13287-020-02009-7

**Published:** 2020-11-25

**Authors:** Chan Chen, Yang Lou, Xin-Yi Li, Zheng-Tian Lv, Lu-Qiu Zhang, Wei Mao

**Affiliations:** 1 Hangzhou Xiaoshan district Hospital of TCM, Jiangnan Hospital Affiliated to Zhejiang Chinese Medical University, Hangzhou, 311201 Zhejiang China; 2 Zhejiang Chinese Medical University, Hangzhou, 310053 Zhejiang China; 3 The first Affiliated Hospital Zhejiang Chinese Medical University, Hangzhou, 311006 Zhejiang China

**Keywords:** Mesenchymal stem cells, Cardiovascular, Knowledge mapping analysis, Visualization

## Abstract

**Background:**

Mesenchymal stem cells (MSCs) have important research value and broad application prospects in the cardiovascular disease. This study provides information on the latest progress, evolutionary path, frontier research hotspots, and future research developmental trends in this field.

**Methods:**

A knowledge map was generated by CiteSpace and VOSviewer analysis software based on data obtained from the literature on MSCs in the cardiovascular field.

**Results:**

The USA and China ranked at the top in terms of the percentage of articles, accounting for 34.306% and 28.550%, respectively. The institution with the highest number of research publications in this field was the University of Miami, followed by the Chinese Academy of Medical Sciences and Harvard University. The research institution with the highest ACI value was Harvard University, followed by the Mayo Clinic and the University of Cincinnati.

The top three subjects in terms of the number of published articles were cell biology, cardiovascular system cardiology, and research experimental medicine. The journal with the most publications in this field was Circulation Research, followed by Scientific Reports and Biomaterials. The direction of research on MSCs in the cardiovascular system was divided into four parts: (1) tissue engineering, scaffolds, and extracellular matrix research; (2) cell transplantation, differentiation, proliferation, and signal transduction pathway research; (3) assessment of the efficacy of stem cells from different sources and administration methods in the treatment of acute myocardial infarction, myocardial hypertrophy, and heart failure; and (4) exosomes and extracellular vesicles research. Tissue research is the hotspot and frontier in this field.

**Conclusion:**

MSC research has presented a gradual upward trend in the cardiovascular field. Multidisciplinary intersection is a characteristic of this field. Engineering and materials disciplines are particularly valued and have received attention from researchers. The progress in multidisciplinary research will provide motivation and technical support for the development of this field.

## Introduction

Mesenchymal stem cells (MSCs) are widely perceived as a class of adult pluripotent stem cells with multiple differentiation potentials, which derived from mesoderm and neuroectoderm and do not express hematopoietic-related markers [1]. The important biological characteristics of MSCs include its low level of expression of human leukocyte antigen class I molecules and CD40, CD40 ligand, CD80, or CD86, which is required to induce effector T cells [2]. MSCs have low immunogenicity and immunoregulatory effects, which can affect every cell of the immune system through cell-cell interactions and paracrine effects [3]. Based on these special biological characteristics, MSCs have important research value and broad application prospects.

Recent studies have shown that the use of MSCs has made great progress in cardiovascular basic and clinical research. MSCs induce the differentiation of cardiomyocytes and vascular endothelial cells. Bonnet found that BMSCs induced in vitro can express platelet-derived growth factor receptor (PDGFR), smooth muscle myosin heavy chain 11 (SMMHC11), and myoglobulin light chain 2 (MLC2), which is similar to that observed in aortic smooth muscle cells, and BMSCs have similar electrophysiological activity and contraction ability [4]. MSCs inhibit myocardial fibrosis; for example, MSC exosomes reduce fibrosis of the heart by inhibiting the proliferation of fibroblasts, promoting the synthesis of metalloproteinases, and stimulating angiogenesis in the infarct area [5]. MSCs also promote angiogenesis. Rahbarghazi transplanted MSCs into infarcted myocardium in rabbits and found that the surrounding area of infarcted myocardium mainly differentiated into cardiomyocytes, endothelial cells, and smooth muscle cells, and the microvessel density significantly increased [6]. MSCs can effectively treat myocardial infarction, dilated cardiomyopathy, heart failure, and other conditions. For example, Lee et al. proved that intravenous injection of bone marrow MSCs was safe, mild, and effective and had a long-lasting effect [7]. Chin et al. confirmed that autologous bone marrow-derived MSCs were safe and effective in treating DCM [8]. Bartunek et al. found that MSC therapy did not produce myocardial toxicity, which significantly increased the left ventricular ejection fraction, reduced the end-systolic volume, and increased the 6-min walking distance of HF patients [9].

Bibliometrics uses the literature system and bibliometric characteristics as the research object and conducts quantitative and qualitative analysis of the literature [10]. It allows the quantitative measurement of the profile distribution as well as the relationships and clustering of studies [11]. In addition to describing and predicting the development of a particular research area, this type of analysis can also compare the contributions of different countries, institutions, journals, and scholars [12]. This type of analysis technology is playing an increasingly important role in developing guidelines and evaluating research trends [13]. Many scholars have used this method of literature analysis in various fields of medicine, such as spinal surgery research [14], health information research [15], biological signaling molecule research [16], neurogenetics research [17], and endocrine disease research [18].

This research is based on data regarding the literature on MSCs in the cardiovascular field, which uses CiteSpace and VOSviewer to form a corresponding knowledge map and recognize the knowledge base. The study provides information on the latest progress, evolutionary path, frontier research hotspots, and future research developmental trends in this field.

## Methods

### Data collection

SCI-E and SSCI of the core database of the document information index database Web of Science were selected as the target databases for source document retrieval. The search formula was set to TS = (“cardiovascular” OR “heart” OR “circulation”) AND TS = (mesenchymal stem cells), and the dates of the search were from January 1, 2010, to March 31, 2020, which resulted in a total of 3455 records. There were 8 types of documents among the 3455 records. As shown in Table 1, there were 2380 articles, which accounted for 72.187% of the total number of records, making articles the most common type of literature. Reviews ranked second, as there were 755 reviews, accounting for 22.900% of the total. The other 8 document types were meeting abstracts (98), editorial materials (55), book chapters (35), proceedings papers (32), early access (20), letters (5), corrections (3), and news items (1).

**Table 1:** Document types of the publications

No.	Type of document	TP	SOTC	CA	Proportion/%	*h*-index
1	Article	2380	54,115	32,037	72.187	89
2	Review	755	25,090	20,693	22.900	77
3	Meeting Abstract	98	16	12	2.972	1
4	Editorial Material	55	411	403	1.668	9
5	Book Chapter	35	946	943	1.062	12
6	Proceedings Paper	32	891	871	0.971	15
7	Early Access	20	5	5	0.607	1
8	Letter	5	8	8	0.152	2
9	Correction	3	7	7	0.091	2
10	News Item	1	0	0	0.030	0

*TP* total publications, *SOTC* sum of times cited, *CA* citing articles

### Data analysis

VOSviewer and CiteSpace were used to analyze the 3384 exported articles. VOSviewer constructs a map based on the cooccurrence matrix. The construction of the map is a three-step process. In the first step, the similarity matrix is calculated based on the cooccurrence matrix. In the second step, the VOS mapping technique is applied to the similarity matrix to construct a map. Finally, in the third step, the map is translated, rotated, and reflected [19]. The term cooccurrence graph in VOSviewer only includes terms that appear in the title and are abstracted at least 50 times under the binary count [19]. The purpose of the algorithm is to ensure that terms that occur more frequently have larger bubble images and that terms with high similarity are close to each other [20]. CiteSpace is a web-based Java application that focuses on detecting and analyzing the evolution of research frontiers and the relationship between research frontiers and their knowledge base. CiteSpace also examines the internal connections between different research frontiers [21]. It is used to capture keywords associated with strong citation bursts, which can be used as predictors of research frontiers.

## Results

### Temporal distribution map of the literature

**Fig. 1:**
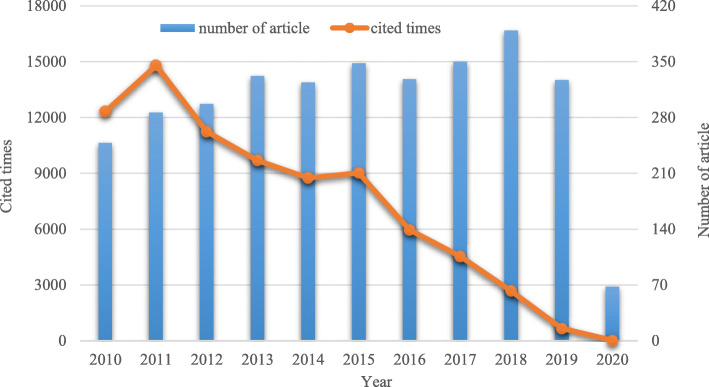
Trends in the growth of publications and the number of cited articles worldwide from 2010 to 2020

From 2010 to 2018, the number of research publications on MSCs in the cardiovascular field generally showed an upward trend (Fig. 1). From 2010 to 2013, the number of articles published in this field rose steadily, with a slight decline in 2014, an increase in 2015, and a decline in 2016. The number of articles increased each year from 2017 to 2018. In 2018, the number of articles reached its peak and then declined in 2019. As shown in Fig. 1, documents published between 2010 and 2015 were cited more frequently, and the most cited articles were published in 2011.

### Country/region distribution

As shown in Table 2, the number of articles published by the USA and China placed them at the top of the ranking, as each accounted for 34.306% and 28.550% of the total, respectively. The total number of studies conducted by both countries comprised more than half of the total, indicating that the two countries had high research interest in this field. The top three countries in terms of ACI values were the Netherlands (40.2288), Spain (34.2771), and the USA (32.5757), suggesting that these three countries had started to conduct research in this field earlier than other countries and that their research results were more mature.

**Table 2:** Top 10 productive countries in regard to the research on mesenchymal stem cells in cardiovascular disease

Rank	Country	Region	Quantity	Percentage	ACI	*H*-index	Total link strength
1	USA	North America	1129	34.306	32.5757	89	541
2	China	East Asia	919	28.550	16.7737	53	258
3	Italy	South Europe	188	5.840	26.766	38	157
4	Germany	Central Europe	184	5.716	32.4293	43	168
5	South Korea	East Asia	144	4.473	21.4861	31	68
6	England	Western Europe	126	3.914	27.2619	32	143
7	Netherlands	Western Europe	118	3.555	40.2288	40	100
8	Canada	North America	116	3.604	28.2586	30	78
9	Japan	East Asia	115	3.573	25.5304	30	66
10	Spain	Southern Europe	83	2.578	34.2771	27	59

*ACI* average citations per item

**Fig. 2:**
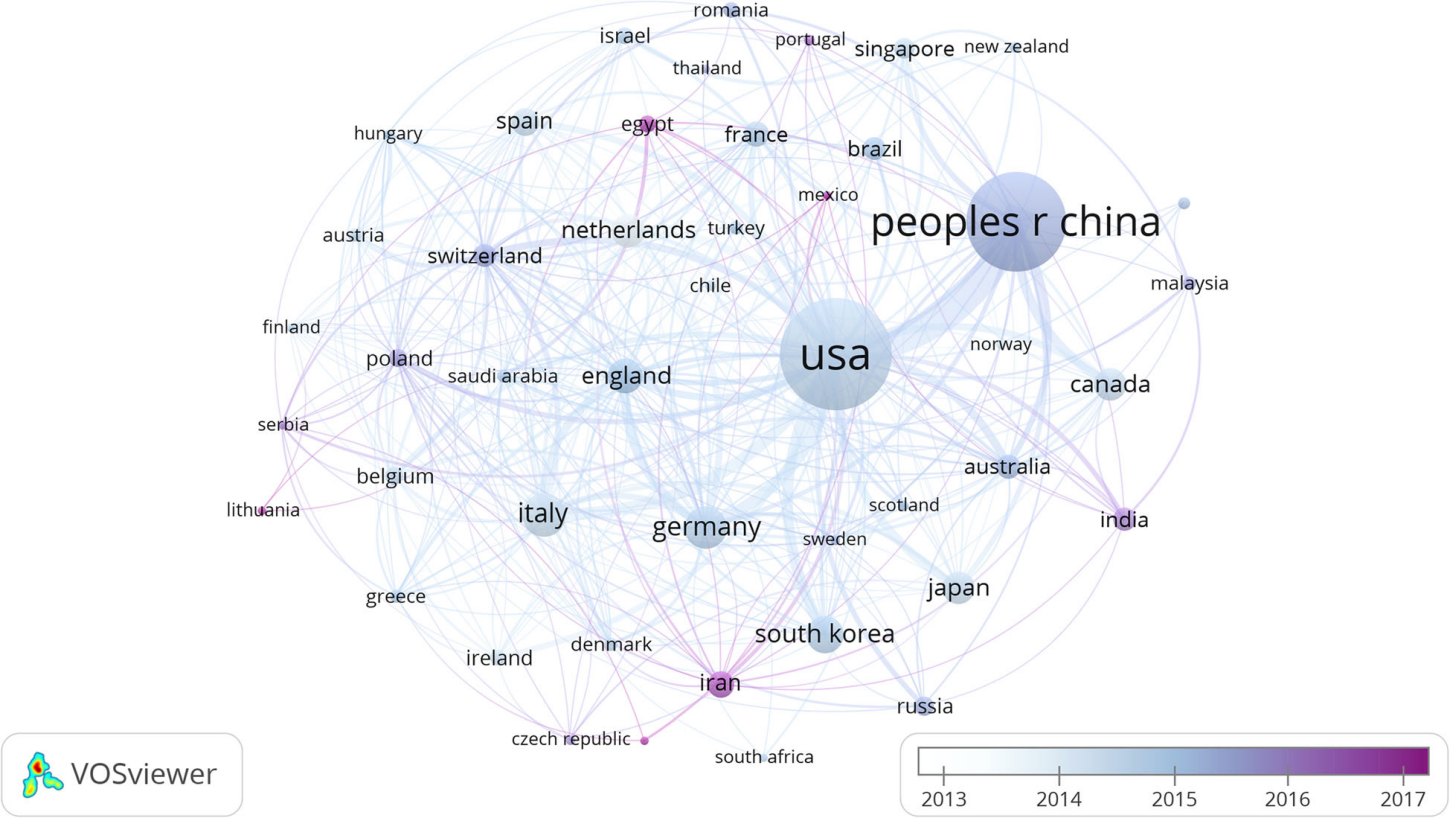
Cooperation map of countries of mesenchymal stem cells in cardiovascular disease

As shown in Fig. 2, countries with close cooperation can be mainly divided into three types. The green part: the USA and China showed the greatest cooperation with South Korea, Japan, Canada, and Australia. The brown part: Germany and Italy worked more closely with England, Netherlands, France, Switzerland, and Spain. The blue part: Singapore contacted with New Zealand and Portugal closely, and Malaysia worked more frequently with India.

### Distribution of authors and research institutions

As shown in Table 3, Hare JM from the University of Miami in the USA has the highest number of published articles, followed by Wang Y from Shanghai Jiao Tong University in China and Zhang Yu from the Second Military Medical University in China. Seven of the top ten writers are from China, and three are from the USA.

**Table 3:** Top 15 authors in the studies of mesenchymal stem cells in cardiovascular disease

Rank	Author	Country	Institute	TP	*P*	*h*
1	Hare JM	USA	Univ Miami	64	1.941%	28
2	Wang Y	China	Shanghai Jiao Tong Univ	58	1.759%	21
3	Zhang, Yu	China	Second Mil Med Univ	42	1.274%	21
4	Zhang, Lei	China	Southeast Univ	39	1.183%	16
5	Ashraf, Muhammad	USA	Augusta Univ	32	0.971%	18
6	Li, Yan	China	Fourth Mil Med Univ	27	0.819%	11
7	Li, Xin	China	Guangdong Acad Med Sci	26	0.789%	11
8	Liu, Yue	China	China Acad Chinese Med Sci	25	0.758%	8
9	Cao, Feng	China	Fourth Mil Med Univ	24	0.728%	16
10	Heldman, Alan W.	USA	Univ Miami	24	0.728%	15

*TP* total publications, *h H*-index

**Fig. 3:**
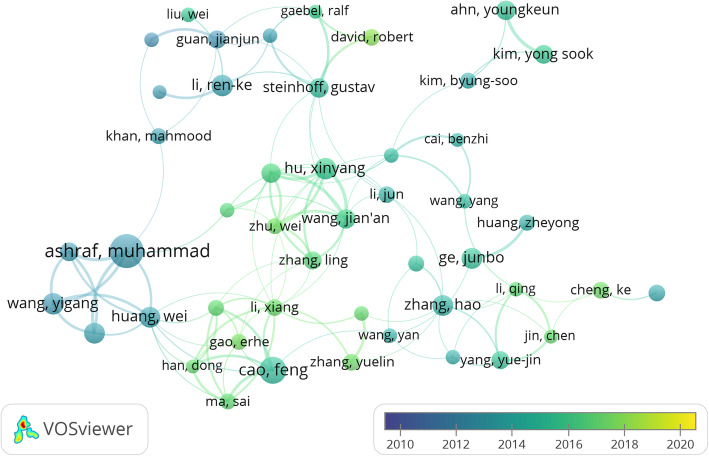
Cooperation map of authors in the studies of mesenchymal stem cells in cardiovascular disease

As shown in Fig. 3, different colors represent clusters of close cooperation. For examples, Ashraf and Muhammad worked closely with Wang Yigang and Huang Wei. Li Ren-ke worked closely with Steinhoff, Gustav, David Robert, Guan Jianjun, Khan Mahmood, and so on.

As shown in Table 4, the institution with the highest number of research publications in this field is the University of Miami with a quantity of 73, followed by the Chinese Academy of Medical Sciences with a quantity of 55 and Harvard University with a quantity of 52. The research institution with the highest ACI value in this field was Harvard University, which had an ACI value of 92.2692, followed by Mayo Clinic with an ACI value of 36.5227 and University of Cincinnati with an ACI value of 34.5455.

**Table 4:** Top 10 institutions in the studies of mesenchymal stem cells in cardiovascular disease

Rank	Institution	Country	Quantity	Total link strength	STC	ACI
1	Univ Miami	USA	73	23	3339	45.7397
2	Chinese Acad Med Sci	China	55	57	1319	23.9818
3	Harvard Univ	USA	52	24	4798	92.2692
4	Sun Yat-sen Univ	China	51	5	626	12.2745
5	Fourth Mil Med Univ	China	48	25	883	18.3958
6	Peking Union Med Coll	China	47	55	1173	24.9574
7	Mayo Clin	USA	44	9	1607	36.5227
8	Univ Cincinnati	USA	44	9	1520	34.5455
9	Univ Toronto	Canada	44	14	1410	32.0455
10	Fudan Univ	China	42	13	762	18.1429

*STC* sum of the times cited, *ACI* average citations per item

**Fig. 4:**
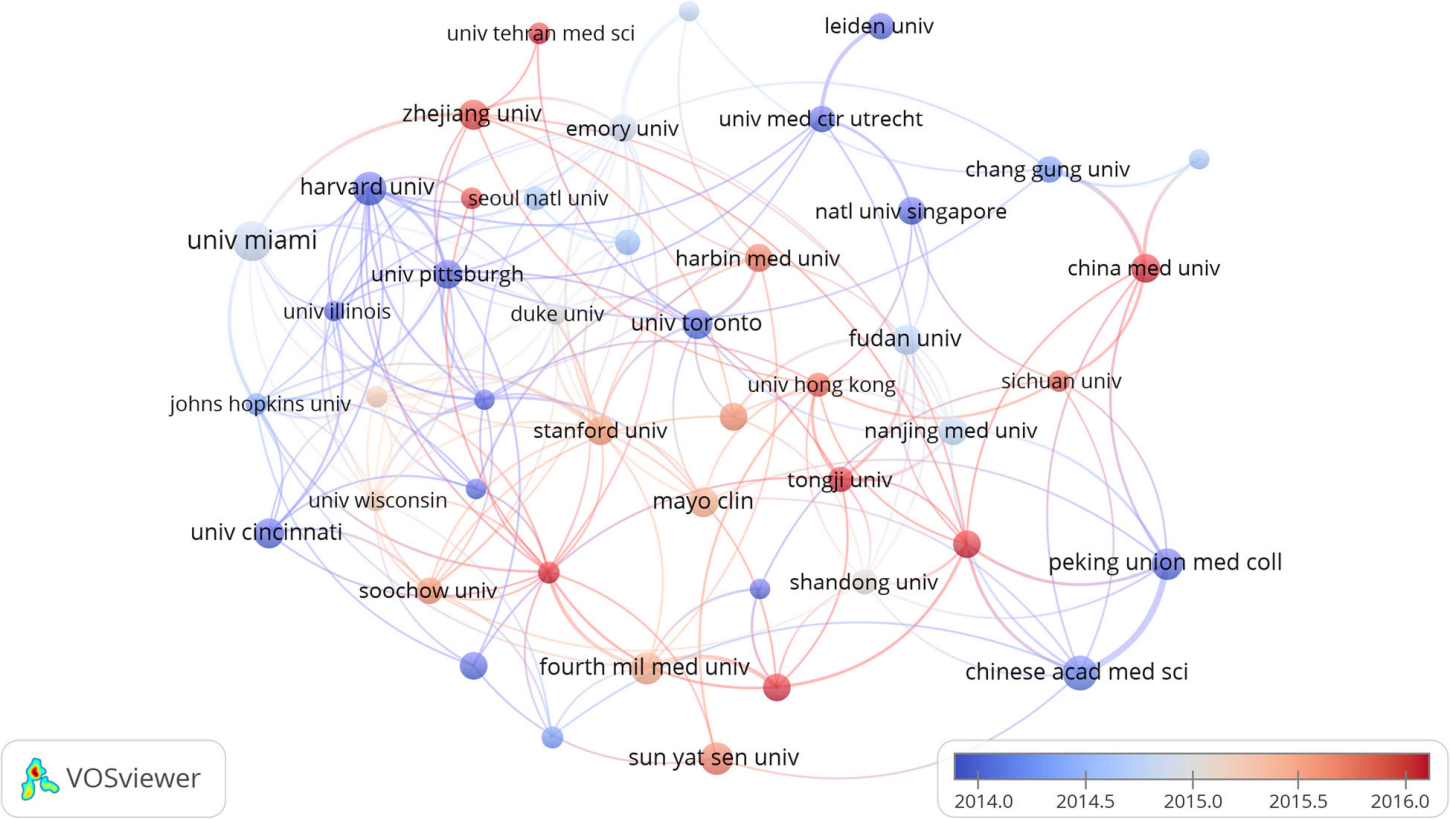
Cooperation map of institutions

As shown in Fig. 4, different colors mean clusters of intimate relationship. The University of Miami cooperated closely with Harvard University, Pittsburgh University, and Zhejiang University. Sun Yat-sen University cooperated closely with Fudan University, and so on.

### Distribution of disciplines in the literature

As shown in Table 5, the top three disciplines in terms of the number of published articles were cell biology (30.664%), cardiovascular system cardiology (20.534%), and research experimental medicine (20.140%). Additional disciplines represented in the literature were engineering (9.827%), materials science (9.160%), biochemistry and molecular biology (8.280%), biotechnology and applied microbiology (7.158%), pharmacology and pharmacy (6.946%), transplantation (4.974%), chemistry (3.063%), and other disciplines, indicating that the research performed in this field was broad and that the research methods were diverse.

**Table 5:** The top 20 subject categories in the studies of mesenchymal stem cells in cardiovascular disease

Rank	Quantity	WOS categories	Percentage
1	1011	Cell biology	30.664
2	677	Cardiovascular system cardiology	20.534
3	664	Research experimental medicine	20.140
4	324	Engineering	9.827
5	302	Materials science	9.160
6	273	Biochemistry and molecular biology	8.280
7	236	Biotechnology and applied microbiology	7.158
8	232	Science technology—other topics	7.073
9	229	Pharmacology and pharmacy	6.946
10	222	Hematology	6.733
11	164	Transplantation	4.974
12	159	Oncology	4.823
13	129	Physiology	3.913
14	123	Surgery	3.731
15	101	Chemistry	3.063
16	96	General internal medicine	2.912
17	80	Endocrinology and metabolism	2.426
18	62	Respiratory system	1.880
19	57	Biophysics	1.729
20	57	Immunology	1.729

As shown in Table 6, the journal with the highest number of articles in this field was Circulation Research, followed by Scientific Reports (42), Biomaterials (40), Molecular Medicine Reports (40), Stem Cells (39), and Tissue Engineering Part A (39). The magazine with the highest ACI value was Biomaterials (17.68), followed by Stem Cells Translational Medicine (17.45), Journal of Cellular and Molecular Medicine (16.48), Circulation Research (15.30), Tissue Engineering Part A (14.8182), Cell Transplantation (14.78), and Scientific Reports (13.9167).

**Table 6:** Top 15 journals in the studies of mesenchymal stem cells in cardiovascular disease

Rank	Journal title	Quantity	ACI
1	Circulation Research	49	15.30
2	Scientific Reports	42	13.9167
3	Biomaterials	40	17.68
4	Molecular Medicine Reports	40	12.4091
5	Stem Cells	39	8.95
6	Tissue Engineering Part A	39	14.8182
7	Stem Cells And Development	28	13.66
8	Cell Transplantation	27	14.78
9	Circulation	25	11.48
10	Journal of Cellular and Molecular Medicine	24	16.48
11	International Journal of Cardiology	22	2.25
12	PLOS One	22	7.02
13	Stem Cell Research & Therapy	18	6.16
14	Stem Cells Translational Medicine	17	17.45
15	Stem Cells International	11	5.15

*ACI* average citations per item

### Highly cited literature analysis

As shown in Table 7, the article “Pericytes: Developmental, Physiological, and Pathological Perspectives, Problems, and Promises” was cited the most often. Armulik discussed the important roles of pericytes in the processes of organismal development and vascular homeostasis and their relationship with MSCs [22].

**Table 7:** Top 15 co-cited articles, cited authors, and cited references

Rank	Title	Journal	Type	Authors	Y	C	IN	CN
1	Pericytes: Developmental, Physiological, and Pathological Perspectives, Problems, and Promises	Developmental Cell	Review	Armulik, Annika.et al.	2011	1086	2	2
2	Comparison of Allogeneic vs Autologous Bone Marrow-Derived Mesenchymal Stem Cells Delivered by Trans	Journal of the American Medical Association	Article	Hare, Joshua M.et al.	2012	639	8	1
3	Whole-Organ Tissue Engineering: Decellularization and Recellularization of Three-Dimensional Matrix	Annual Review of Biomedical Engineering	Review	Badylak, Stephen F.et al.	2011	516	7	1
4	Bone Marrow Mesenchymal Stem Cells Stimulate Cardiac Stem Cell Proliferation and Differentiation	Circulation Research	Article	Hatzistergos, Konstantinos E. et al.	2010	469	5	2
5	Molecular mechanisms of cancer development in obesity	Nature Reviews Cancer	Review	Khandekar, Melin J. et al.	2011	467	4	1
6	Harnessing the Mesenchymal Stem Cell Secretome for the Treatment of Cardiovascular Disease	Cell Stem Cell	Review	Ranganath, Sudhir H. et al.	2012	449	6	2
7	Conversion of vascular endothelial cells into multipotent stem-like cells	Nature Medicine	Article	Medici, Damian et al.	2010	444	8	1
8	Mesenchymal stem cell-derived exosomes increase ATP levels, decrease oxidative stress and activate P	Stem Cell Research	Article	Arslan, Fatih Et al.	2013	436	8	2
9	Clinical Trials With Mesenchymal Stem Cells: An Update	Cell Transplantation	Review	Squillaro, Tiziana et al.	2016	411	4	3
10	Aggregation of human mesenchymal stromal cells (MSCs) into 3D spheroids enhances their antiinflammat	Proceedings of the National Academy of Sciences of the United States of America	Article	Bartosh, Thomas J. et al.	2010	411	1	1
11	Perivascular Gli1(+) Progenitors Are Key Contributors to Injury-Induced Organ Fibrosis	Cell Stem Cell	Article	Kramann, Rafael. et al.	2015	337	6	2
12	Cell Therapy for Heart Failure: A Comprehensive Overview of Experimental and Clinical Studies, Curren	Circulation Research	Review	Sanganalmath, Santosh K. et al.	2013	332	2	1
13	Immunosuppressive Properties of Mesenchymal Stem Cells: Advances and Applications	Current Molecular Medicine	Review	De Miguel, M. P. et al.	2012	327	4	1
14	The war against heart failure: the Lancet lecture	Lancet	Review	Braunwald, Eugene.	2015	301	2	1
15	Bone Marrow-Derived Cell Therapy Stimulates Endogenous Cardiomyocyte Progenitors and Promotes Cardia	Cell Stem Cell	Article	Loffredo, Francesco S. et al.	2011	292	2	1

*Y* year, *C* citations, *IN* institute number, *CN* country number

The second most cited article was “Comparison of Allogeneic vs Autologous Bone Marrow-Derived Mesenchymal Stem Cells Delivered by Transendocardial Injection in Patients with Ischemic Cardiomyopathy: The POSEIDON Randomized Trial”. In this article, Hare et al. confirmed that intracardiac injection of allogeneic and autologous MSCs could treat ischemic cardiomyopathy effectively and relatively safely [23].

The third most cited article was “Whole-Organ Tissue Engineering: Decellularization and Recellularization of Three-Dimensional Matrix”. In this article, Badylak explained that the combination of three-dimensional bioscaffold materials and cell transplantation was a promising tissue engineering strategy and a method for the regeneration of functional organs for medical replacement [24]. The above articles could be regarded as constituting an important theoretical basis and providing clinical evidence for research in this field.

According to the types of the articles, 8 of the most highly cited articles were reviews, and 7 were monographs. Based on the publication dates of the most highly cited articles, the most highly cited articles were published from 2011 to 2013, followed by 2015 to 2016. These periods can be regarded as representing the two stages of the development of this field. Based on the numbers of cooperating institutions and countries, there were 10 articles involving more than three institutions and 6 articles involving groups in at least two countries.

### Research hotspots and frontier analysis

#### Research hotspot analysis

Keywords reflect the core content of the article and can be used to identify the evolving research frontiers related to the knowledge field [25]. As shown in Table 8, in addition to mesenchymal stem cells and heart, the keywords with a high frequency of occurrence were heart transplantation (582), differentiation (535), myocardial infarction (482), in vitro (473), therapy (472), and progenitor cells (458).

**Table 8:** The top 20 keywords in the studies of mesenchymal stem cells in cardiovascular disease

Rank	Keywords	Occurrences	Total link strength	Rank	Keywords	Occurrences	Total link strength
1	Mesenchymal stem cells	1529	6487	11	Myocardial infarction	456	3032
2	Heart	594	3166	12	Bone marrow	449	2468
3	Transplantation	582	3386	13	Expression	362	1782
4	Mesenchymal stem cells	561	3066	14	Acute myocardial infarction	331	1985
5	Differentiation	535	2780	15	Angiogenesis	304	1783
6	Stromal cells	486	2628	16	Regeneration	290	1814
7	Myocardial infarction	482	2707	17	Stem cells	283	1674
8	In vitro	473	2476	18	Cardiomyocytes	273	1603
9	Therapy	472	2708	19	Heart failure	273	1529
10	Progenitor cells	458	2634	20	Repair	272	1683

**Fig. 5:**
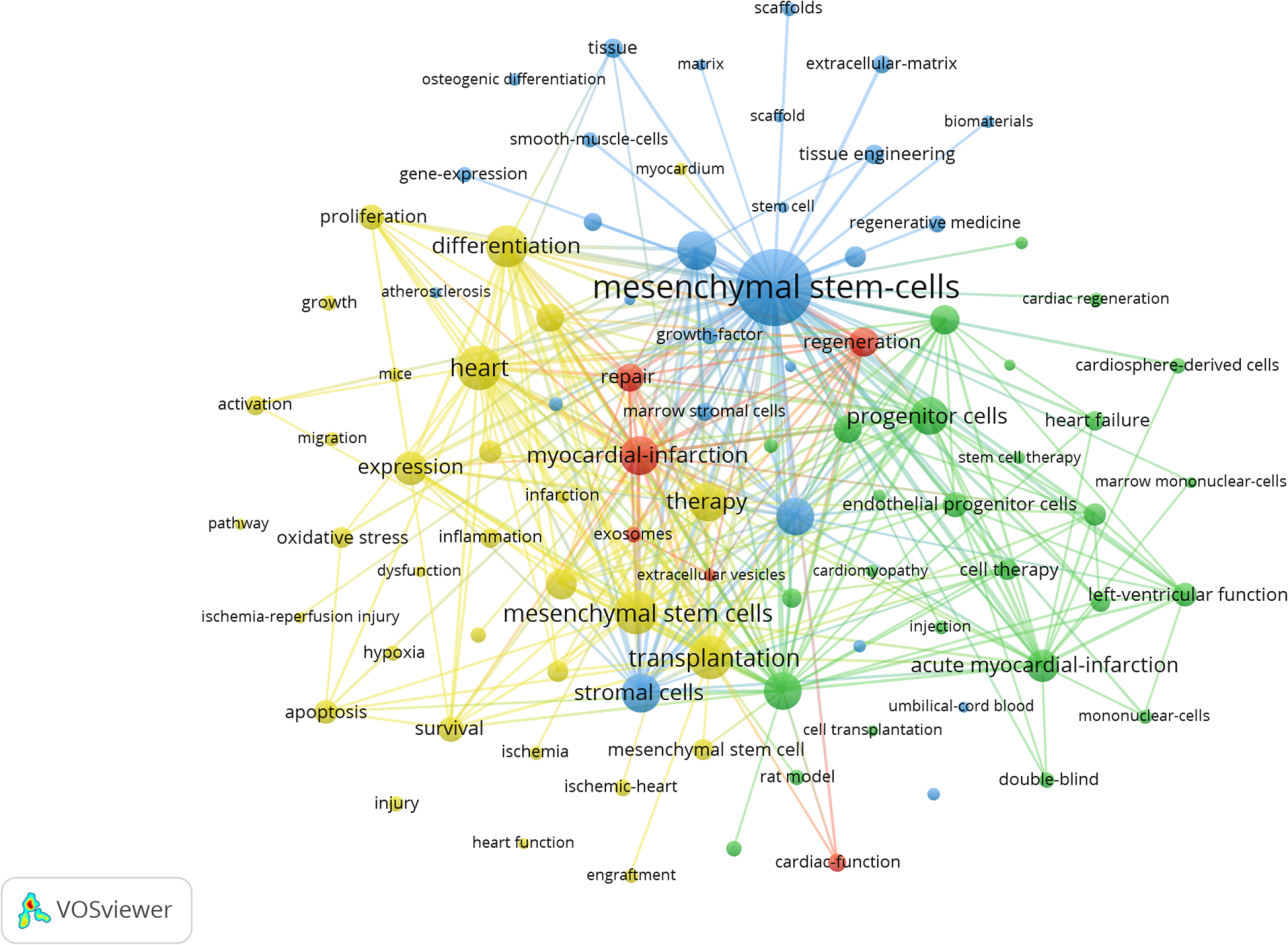
Map of keyword clustering in the studies of mesenchymal stem cells in cardiovascular disease

As shown in Fig. 5, in the keyword cooccurrence network map, the thicker the connection between the nodes is, the more frequently the two keywords appear together. The keywords formed 4 clusters, which represented the four major research directions in the field.

The blue cluster was predominated by tissue engineering, scaffolds, and extracellular matrix. Tissue engineering researched the potential efficacy of HTMSCs [26], ADMSCs [27], and HUCMSCs [28] in repairing myocardial tissue. It studied elastic polyurethane nanofiber [29], copolymerization material [30], nano cellulose patch [31], and 3D biomaterial [32] to promote integration of MSCs and myocardial tissue. Scaffolds explored nanofiber scaffold [33, 34], collagen scaffold [35], alginate scaffold [36], biomatrix scaffolds [36, 37], and porous polysaccharide-based scaffold [38]. Extracellular matrix discussed decellularized bovine myocardial extracellular matrix-based films(dMEbF) [39], synthetic extracellular matrix mimic hydrogel [40], cardiac fibroblast-derived 3D extracellular matrix [41], and porcine small intestinal submucosal extracellular matrix [42] enhanced functions of MSC.

The yellow cluster was mainly composed of transplantation, differentiation, proliferation, oxidative stress, inflammation, and apoptosis. The first three parts researched regulatory factors [43–46], drugs [47–49], and tracking tools [50–52], which improve efficacy of MSCs in transplantation, differentiation, and proliferation. The last three parts studied mechanisms of MSCs in antioxidant, anti-inflammatory, and anti-apoptotic. The mechanisms contain effects of inducible factor and drugs, such as high density lipoprotein (HDL) [53], telomere repeat binding factor 2 interacting protein 1 (terf2ip), Asprosin [54], Nicorandil [55], Metformin [56], and artemisinin [57].

The green cluster focused on marrow mononuclear cells, endothelial progenitor cells, acute myocardial infarction, left ventricular function, and double blind. Marrow mononuclear cells researched the comparison of MSCs and BMSCs in safety of intracardiac injection [58, 59]. Endothelial progenitor cells investigated effect of MSCs and EPCs in inducing vascular smooth muscle and promoting angiogenesis [60, 61]. Acute myocardial infarction and left ventricular function discussed myocardial protective mechanism of MSCs. MiR-133 [62], PKC ɛ [63], and glucagon-like peptide-1 [64] could improve MSCs function. The enhancement of left ventricular systolic function by MSCs mainly occurred in the anterior myocardial segment near the infarcted area of LAD [65]. Double blind evaluated the clinical efficacy of MSCs in heart failure treatment by intravenous infusion [66], intramyocardial injection [67], and endocardial transport [68].

The main research topics of red cluster were exosomes and extracellular vesicles. They could mediate mir-21-5p [69], mir-223 [70], mir-26a [71], mir-210 [72], and mir-19a [73], which enhanced MSCs to play a role in angiogenesis, myocardial cell viability, and myocardial protection.

#### Combined evolutionary path

**Fig. 6:**
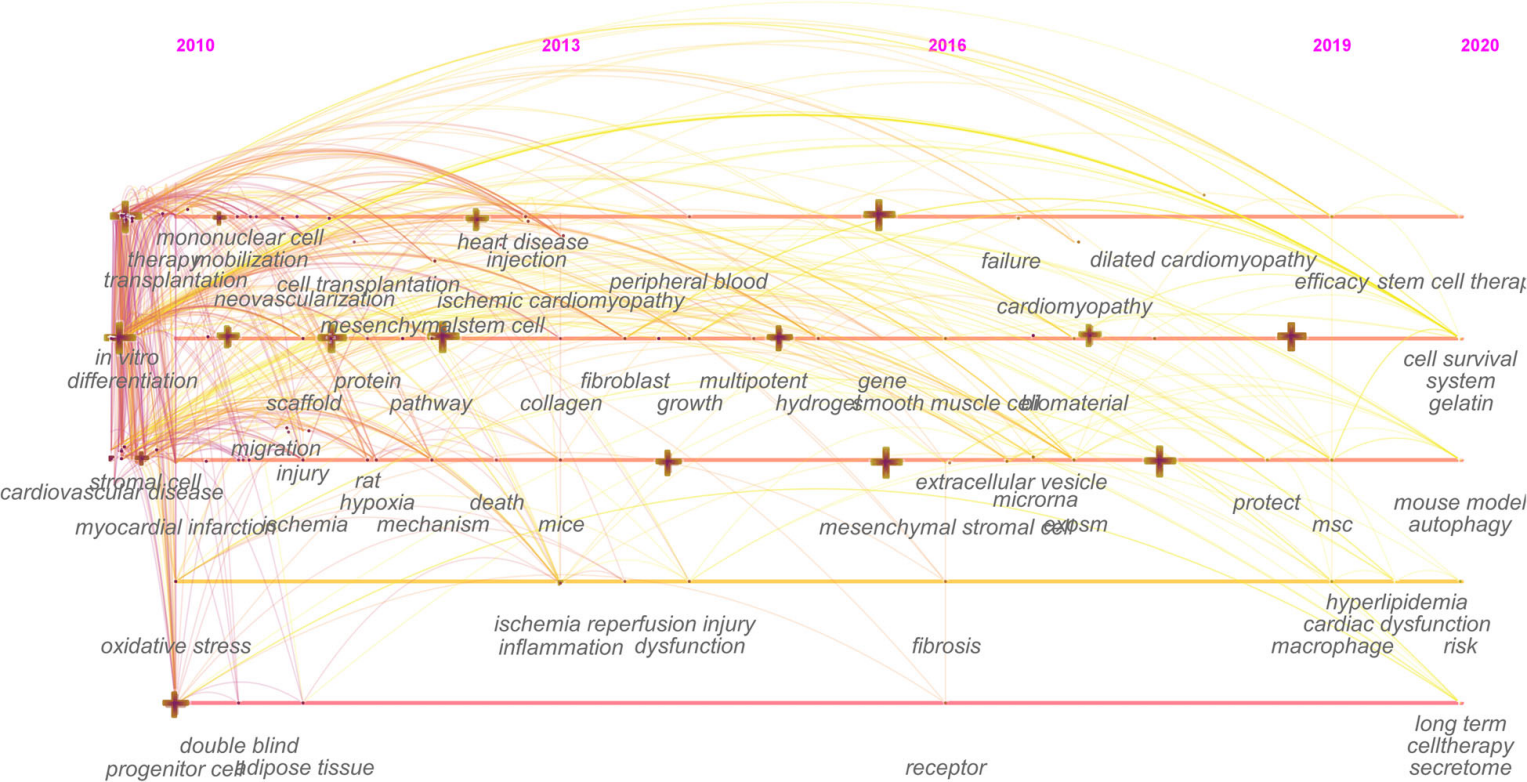
Evolutionary path in the studies of mesenchymal stem cells in cardiovascular disease

In Fig. 6, the year corresponding to each of the keywords is the first year it appeared in the analyzed data set. The transformation between nodes could reveal the evolution of MSCs in the cardiovascular research hotspot. From 2010 to 2012, cardiovascular MSC research began to focus on apoptosis, left ventricular function, proliferation, bone marrow cells, and endothelial cells. In 2013–2015, endothelial progenitor cells, extracellular matrix, ischemic cardiomyopathy, and tissue received increased attention in the field. From 2016 to 2017, the field turned to research on injury and oxidative stress. Tissue engineering, exosomes, and inflammation became the new focus in 2018–2020.

#### Research frontier identification

In Table 9, the timeline is depicted as a blue line, while burst detection is shown as a red segment on the blue timeline that indicates the start year, end year, and duration of the burst. In particular, we are interested in the key words with research significance, which reflect the evolutionary trend of this field.

**Table 9:**
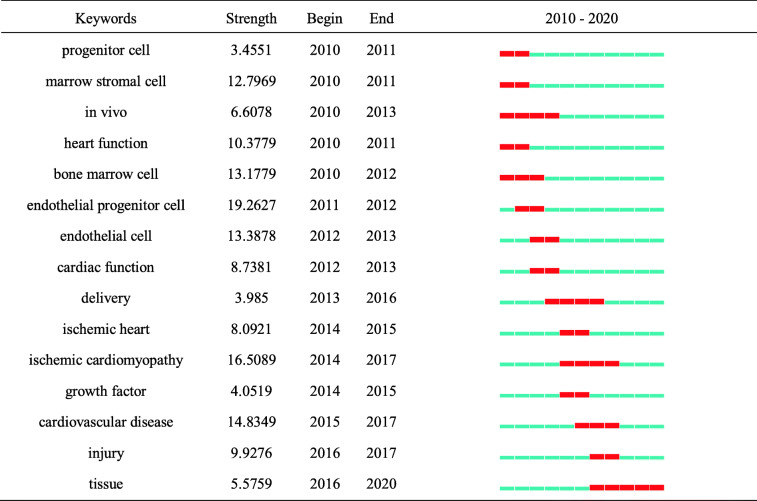
Top 15 keywords with the strongest citation bursts

Endothelial progenitor cells showed the strongest burst strength, followed by ischemic cardiomyopathy, cardiovascular disease, and endothelial cells. The terms progenitor cells, marrow stromal cells, and heart function appeared for the first time recently but persisted for a shorter period of time. The burst times of the terms delivery, ischemic heart, and ischemic cardiomyopathy were consistent. Tissue is the current research frontier in this field and is currently within the burst period.

## Discussion

This paper used the information visualization software CiteSpace and VOSviewer to carry out bibliometric analysis of the literature on MSCs in the cardiovascular field published from 2000 to 2020. The analysis assessed the spatial and temporal distribution, author contribution, core literature, research hotspots, and research frontier analysis. We used keyword cooccurrence analysis to identify research hotspots in each period and to determine the core evolutionary path of the theme. Then, we identified the current research frontiers of research of MSCs in the cardiovascular field. The main conclusions are as follows:

1. The research of MSCs in the cardiovascular field showed a zigzag upward trend.

Stem cell therapy has great potential for use in future regenerative medicine treatment; however, it has some risks and limitations, such as the type of stem cells used, cell proliferation ability, differentiation status, drug delivery route, drug delivery site, and the ability for survival of transplanted cells, which will affect the therapeutic effect. The therapeutic characteristics, medical ethics, and possible teratogenicity have made the study of MSCs highly controversial [74]. For example, Hegyi et al. proposed that MSCs might form primary cardiac sarcomas and develop into tumors with multiple lineage differentiation [75]. Huang et al. reported that transplantation and differentiation of MSCs led to progressive ventricular dysfunction and other diseases [76]. In view of the risks of research and the limitations of technology, researchers are cautious in carrying out the research work.

(2) The study of cardiovascular MSCs in Europe and America started first, and Asian countries have paid increasing attention to this area of research.

European and American countries started research in this field earlier than other countries, and their research is therefore more mature. For example, Harvard University, the Mayo Clinic, and University of Cincinnati in the USA have obtained a large number of high-quality research results. Harvard University has mainly studied 3D microcapsules [77] and engineered three-layer scaffolds [78]. The Mayo Clinic has explored the effect of MSCs on left ventricular assist device (LVAD) implantation [79], left ventricular remodeling [80], and heart failure [80] through clinical trials while developing microfiber stents [81] and vascular biomaterials [82]. The paracrine effect of nuclear casein kinase on MSC and MSC-derived extracellular vesicles (EVs) has been studied [83]. The University of Cincinnati explored the cardiac protection mechanism of paracrine MSCs, which involves iPS cells (MiPS) [84], the Wnt11 signaling pathway [85], CXCR4 factor expression [86], the suicide gene [87], and the clusterin Akt/GATA-4 pathway [88].

For the past decade, Asian countries have also paid attention to MSC research. For example, the Chinese Academy of Medical Sciences and Sun Yat-sen University in China are the leading research institutions with high achievements in the field. The Chinese Academy of Medical Sciences examined the time-distribution characteristics of MSCs in the myocardium and other organs [89]. The mechanism by which statins and Chinese medicines regulate SDF-1/CXCR4 [90], JAK-STAT [91], RhoA/ROCK [48], and AMPK/eNOS [92] in MSCs has been studied. The Chinese Academy of Medical Sciences also explored the improvement of cardiac function by MSCs regulated by matrix-derived factor 1 (SDF1a) [93] and CXC chemokine receptor 4 (CXCR4) [94]. Sun Yat-sen University researched the effects of exons (Exo) [95], the TGF-β superfamily [96], the long noncoding RNA brave heart (lncRNA-Bvht) [97], apelin [98], and granulocyte colony stimulating factor (G-CSF) [99] on the proliferation, differentiation, and vascularization of MSCs. The functions of the transcription factors islet-1 (ISL1) [100] and platelet fibrin (PRF) [101] in regulating the repair of myocardial infarction by MSCs have been explored. Although basic research on cardiovascular stem cells in China is at the forefront of efforts around the world, progress on clinical studies in China is stagnant, which may be related to the lack of efficient scientific approval systems and strict regulatory policies.

(3) Multidisciplinary intersection provides power and technical support for the development of this field.

The literature published in this field is mainly focused on cell biology, cardiovascular system cardiology, and research experimental medicine, as well as engineering, materials science, chemistry, biophysics, and other disciplines, which reflects that multidisciplinary intersection is a characteristic of research in this field. Engineering and materials disciplines, such as bioengineering [102], tissue engineering [103], genetic engineering [104, 105], and biomaterials [106], have received special attention from scholars. The development of these related disciplines will aid in breaking through the limitations of the technical conditions of research in this field.

(4) Tissue research is a hot spot and frontier area in this field.

Tissue research refers to tissue repair and tissue engineering. Heart failure caused by ischemic and nonischemic cardiomyopathy is due to the progressive and complex process of myocardial remodeling. Local compensatory changes at the genetic, molecular, cellular, and interstitial levels are accompanied by ventricular dilatation and the impairment of systolic and diastolic function. The consequence is that billions of cardiomyocytes replaced by fibrous tissue, and the cardiomyocytes and vascular cells are severely injured [107]. Although it has been confirmed that the adult heart contains a small number of active circulating cells, resident stem cells, and progenitor cells [108], its inherent ability for self-regeneration is unable to compensate for the loss in cell quality due to heart failure. At present, heart transplantation is the only treatment that can completely restore heart function for patients with advanced heart failure. However, due to the shortage of donors, this treatment is often limited. The need to cell therapy has aroused great interest in regenerative medicine.

MSCs have advantages in differentiating into cardiomyocytes [109], protection of cardiac muscle cells from apoptosis [110], promotion of vascular endothelial cell recovery [111], angiogenesis [112], and myocardial tissue repair [113], which is the hot spot of cardiovascular regeneration medicine research.

Because MSCs have low retention rate and poor survival rate, they need to be combined with various forms of bioactive tissue structure and effectively integrated into target tissue, which in order to regenerate cardiac tissue and improve cardiac function, such as polyurethane patch [114], elastic mold [115] based on type I collagen and matrix gel, fibrin patch [116], and Graphene [117]. Tissue engineering technology is the key strategy to repair myocardial tissue.

## Conclusion

MSCs have important research value and broad application prospects in the field of cardiovascular. With the help of information visualization technology, we have obtained a more in-depth understanding of the study progression, evolutionary path, frontier hot spots, and future trends of the research of MSCs in cardiovascular disease over the past 10 years. Multinational cooperation and multidisciplinary intersection are the characteristics and trend of the development in the field, and tissue engineering will be the focus of future research.

## Data Availability

The datasets used and/or analyzed during the current study are available from the corresponding author on reasonable request.

## Abbreviations

ADMSCs: The adipose tissue-derived mesenchymal stem cells
AMPK: AMP-activated protein kinase
CD: Cluster of differentiation
CXCR4: The chemokine CXC motif receptor 4
DCM: Dilated cardiomyopathy
eNOS: Endothelial NO synthase
HF: Heart failure
HTMSCs: Human thymus-derived mesenchymal stem cells
HUCMSCs: Human umbilical cord mesenchymal stem cells
iPS: Induced pluripotent stem
JAK: Janus kinases
LAD: Left anterior descending artery
PKC: Protein kinase C
RhoA: Ras homolog family member A
ROCK: Rho-kinase
SDF-1: Stromal cell derived factor-1
STAT: Signal transducer and activator of transcription
